# The release of cardioprotective humoral factors after remote ischemic preconditioning in humans is age- and sex-dependent

**DOI:** 10.1186/s12967-018-1480-0

**Published:** 2018-04-27

**Authors:** André Heinen, Friederike Behmenburg, Aykut Aytulun, Maximilian Dierkes, Lea Zerbin, Wolfgang Kaisers, Maximilian Schaefer, Tanja Meyer-Treschan, Susanne Feit, Inge Bauer, Markus W. Hollmann, Ragnar Huhn

**Affiliations:** 1 Institute of Cardiovascular Physiology, Heinrich-Heine-University Düsseldorf, Universitätsstr. 1, 40225 Düsseldorf, Germany; 2 Department of Anesthesiology, University Hospital Düsseldorf, Moorenstr. 5, 40225 Düsseldorf, Germany; 3 Department of Neurology, University Hospital Düsseldorf, Moorenstr. 5, 40225 Düsseldorf, Germany; 4 Department of Anesthesiology, Laboratory of Experimental Intensive Care and Anesthesiology (L.E.I.C.A.), Academic Medical Center (AMC), University of Amsterdam, Meibergdreef 9, 1105 Amsterdam, The Netherlands

**Keywords:** Cardioprotection, Remote ischemic preconditioning, Age, Sex, Reperfusion injury

## Abstract

**Background:**

Preclinical and proof-of-concept studies suggest a cardioprotective effect of remote ischemic preconditioning (RIPC). However, two major clinical trials (ERICCA and RIPHeart) failed to show cardioprotection by RIPC. Aging and gender might be confounding factors of RIPC affecting the inter-organ signalling. Theoretically, confounding factors might prevent the protective potency of RIPC by interfering with cardiac signalling pathways, i.e. at the heart, and/or by affecting the release of humoral factor(s) from the remote organ, e.g. from the upper limb. This study investigated the effect of age and sex on the release of cardioprotective humoral factor(s) after RIPC in humans.

**Methods:**

Blood samples were taken from young and aged, male and female volunteers before (control) and after RIPC (RIPC). To investigate the protective potency of the different plasma groups obtained from the human volunteers, isolated perfused hearts of young rats were used as bioassay. For this, hearts were perfused with the volunteer plasma (0.5% of coronary flow) before hearts underwent global ischemia and reperfusion. In addition, to characterize the protective potency of humoral factor(s) after RIPC to initiate protection not only in young but also aged hearts, plasma from young male volunteers were transferred to isolated hearts of aged rats. At the end of the experimental protocol, infarct sizes were determined by TTC-staining (expressed as % of left ventricle).

**Results:**

RIPC plasma of young male volunteers reduced infarct size in young rat hearts from 47 ± 5 to 31 ± 10% (p = 0.02). In contrast, RIPC plasma of aged male volunteers had no protective effect. Infarct size after application of control plasma of young female volunteers was 33 ± 10%, and female RIPC plasma did not lead to an infarct size reduction. RIPC plasma of old female initiated no cardioprotection. RIPC plasma of young male volunteers reduced infarct size in isolated hearts from aged rats (41 ± 5% vs. 51 ± 5%; p < 0.001).

**Conclusions:**

The release of humoral factor(s) into the blood after RIPC in humans is affected by both age and sex. In addition, these blood borne factor(s) are capable to initiate cardioprotection within the aged heart.

**Electronic supplementary material:**

The online version of this article (10.1186/s12967-018-1480-0) contains supplementary material, which is available to authorized users.

## Background

Cardiovascular disease (CVD) with ischemic heart disease as the main contributor is the major cause of death worldwide, accounting for more than 30% of all deaths each year [1]. Therefore, it is of tremendous interest to reduce the consequences of CVD, i.e. myocardial ischemia reperfusion (I/R) injury and its subsequent morbidity and mortality. Transient ischemic episodes of peripheral tissue increases the tolerance of the myocardium against a subsequent I/R injury [2]. This phenomenon is called remote ischemic preconditioning (RIPC). RIPC is considered to be an easy to use and inexpensive technique for protecting the heart against I/R injury in many clinical situations [3]. For example, a recent meta-analysis of 13 randomized clinical trials showed a benefit of RIPC in patients with acute myocardial infarction [4]. Although RIPC has been shown to be effective in both animal studies and humans, there are increasing concerns of its protective potential in the clinical arena. To date, two very recent, large-scale clinical trials (ERICCA and RIPHeart) failed to show the effectiveness of RIPC in heart surgery patients [5, 6]. The reason for this discrepancy remains unclear, but there is strong evidence that cardioprotective interventions, e.g. ischemic preconditioning, are affected by numerous factors including age and sex [7, 8]. However, there is only limited information whether both aging and sex also interfere with the protective potential of RIPC. With respect to aging, a sub-group analysis of the ERICCA trial did not indicate age as confounding factor for RIPC [5]. In contrast, a meta-analysis of Zhou et al. showed a reduced incidence of acute kidney injury after RIPC, and this effect was more pronounced in younger patients [9]. In addition, our group demonstrated recently, that the protective effect of RIPC is lost in the aged heart using an experimental in vivo rat model [10]. However, it is unclear which mechanisms might be accountable for the potential loss of protection by RIPC. On the one hand, there might be disorders in signal transduction between the remote organ and the effector organ (i.e. a modification in the release of the humoral factors); on the other hand, there might be structural changes of the protecting signalling pathway within the heart leading to possible age- and sex-dependent loss of cardioprotection by RIPC. To gain insight into the mechanism by which aging, and possibly sex, interfere with RIPC, we used a mixed experimental approach with RIPC induction in humans and the isolated heart model as bioassay to test the hypothesis that the release of humoral factor(s) after RIPC is affected both increasing age and sex.

## Methods

All investigations were conducted after approval of the Local Ethics Committee (# 3911) and Animal Care Committee (O27/12) of the University of Düsseldorf, Germany.

### RIPC in volunteers

After written informed consent blood samples were taken from male and female volunteers of different age. Young volunteers were between 18 and 30 years of age, old volunteers were aged between 60 and 80 years. In total, 40 volunteers were included in the study. Further criteria for inclusion and criteria for exclusion are listed in Table 1.

**Table 1:** Criteria for inclusion and exclusion

Criteria for inclusion	Written informed consent Age: 18–30 or 60–80 years, respectively Adequate age-based response to physical stress Normal performance of upper limbs
Criteria for exclusion	Missing consent Long-term medication Acute medication (within the past 14 days) Peripheral arterial disease Diabetes mellitus Hypertension, NYHA > II Pre-existing nerve damage of the upper limb Status after thrombo-embolic events Smoking (within the past 5 years, > 10 pack years) Pregnancy Chronic pain disorders and psychiatric or neurologic disorders leading to missing legal competence

RIPC was induced by three periods of 5 min of ischemia of one upper arm each followed by 5 min of reperfusion. Ischemia was induced by inflating a blood pressure cuff to 200 mmHg and reperfusion started by deflating the cuff. 50 ml whole blood was taken (lithium heparin for anticoagulation) from the cubital vein of the opposite arm 5 min before and after RIPC (control and RIPC, respectively). Plasma was separated by centrifugation and was stored at − 80 °C until further use. Four groups of volunteers (n = 10 per group) were treated according to this protocol: (1) young male, (2) young female, (3) aged male and (4) aged female volunteers, respectively.

### Infarct size experiments

All in vitro experiments were performed in isolated hearts of 120 young (aged 2–3 months) and 20 old (aged 22–23 months) male Wistar rats. Animals were housed on a 12:12 light/dark schedule with free access to standard chow and water. Rats were anesthetized by intraperitoneal injection of pentobarbital (80 mg/kg body weight) and thoracotomized. Hearts were quickly excised, mounted on a Langendorff system and were perfused with Krebs–Henseleit solution containing (in mM) 118 NaCl, 4.7 KCl, 1.2 MgSO_4_, 1.2 KH_2_PO_4_, 25 NaHCO_3_, 0.5 EDTA, 2.25 CaCl_2_, 11 glucose and 1 lactate at 37 °C and a constant pressure of 80 mmHg. A small fluid-filled balloon was inserted into the left ventricle and left-ventricular end-diastolic pressure (LVEDP) was set at 1–4 mmHg. Throughout experiments heart rate, left ventricular pressure (LVP), and coronary flow were measured continuously and digitized using an analogue to digital converter (PowerLab/8SP, ADInstruments Pty Ltd, Castle Hill, Australia) at a sampling rate of 500 Hz. Phasic LVP was calculated as maximal left ventricular pressure–minimal left ventricular pressure. The data were continuously recorded on a personal computer using Chart for Windows v5.0 (ADInstruments Pty Ltd, Castle Hill, Australia). Arrhythmic intervals were not used for data analysis.

**Fig. 1:**
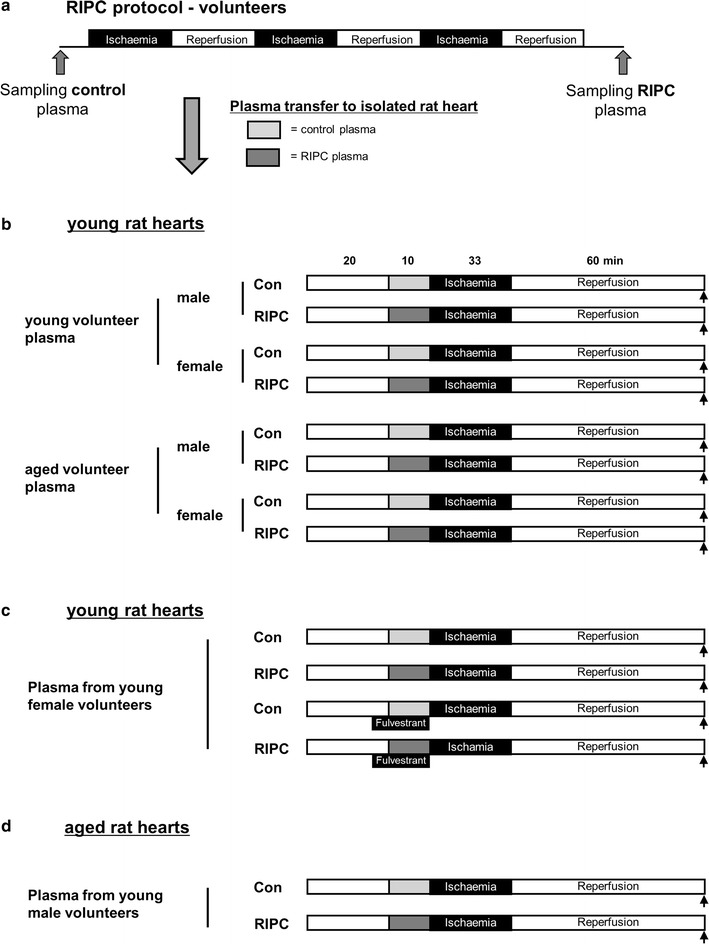
Study design. **a** Schematic diagram of the experimental protocol for induction of RIPC and blood plasma sampling in young and aged, male and female volunteers. RIPC was induced by three periods of 5 min of ischemia of one upper arm each followed by 5 min of reperfusion. **b**–**d** Experimental protocol for isolated heart experiments. Blood plasma samples were transferred to isolated hearts from young (2–3 months) and aged (22–23 months) rat hearts. Black arrows indicate end of reperfusion and infarct size staining. *Con* control, *RIPC* remote ischemic preconditioning

Figure 1 shows the experimental protocol. The experiments were conducted in three independent series. The investigators were blinded for the experimental protocol. In addition, a proper randomization was ensured by determination of a random experimental order by the blinded investigators before the start of the experiments. All hearts of all series (n = 10 hearts per group) underwent an equilibration period of 20 min hearts. The hearts were randomly assigned to one experimental group. In series 1, the effects of the different plasma samples on infarct size were investigated using isolated young male rat hearts as bioassay (Fig. 1b). Hearts from young male animals were used because it has been shown that these hearts are not resistant to conditioning interventions. Prior to ischemia, hearts were perfused with plasma from volunteers for 10 min at a perfusion rate of 0.5% of the coronary flow. Subsequently, hearts were subjected to global ischemia of 33 min, followed by 60 min of reperfusion. At the end of reperfusion, hearts were frozen at − 20 °C for later infarct size staining.

It has been shown that estrogen receptors (ERα and ERβ) are expressed in cardiomyocytes and are involved in acute myocardial protection following ischemia [11, 12]. To test for a potential interference between cardioprotection by RIPC (through humoral factor) and by plasma from young female volunteers (through estrogen), the non-selective estrogen receptor inhibitor fulvestrant (100 nM) was administered 15 min prior to ischemia using isolated hearts from young male rats as bioassay in series 2 (Fig. 1c). At the end of reperfusion, hearts were frozen at − 20 °C for later infarct size staining.

To investigate whether the loss of the cardioprotective potency of RIPC with increasing age is solely caused by defects in the release of humoral factor(s) after RIPC or whether additional cardiac alterations are involved, we transferred control and RIPC plasma from young male volunteer to isolated hearts from aged rats. Plasma from young male volunteers were chosen because it has been shown that the transfer of RIPC plasma from this group initiates protection indicating that humoral factor(s) are released into the blood (Fig. 1d). At the end of reperfusion, hearts were frozen at − 20 °C for later infarct size staining.

### Infarct size staining

The frozen hearts were cut into 1 mm thick slices and stained in 0.75% 2,3,5-triphenyltetrazolium chloride (TTC) for 15 min at 37 °C. Subsequently, slices were fixed in 3.75% paraformaldehyde for 2 h. The fixated slices were scanned and infarct size was analysed by computerized planimetry (Sigma Scan Pro, Version 5, SYSTAT Software, San Jose, California). The investigators were blinded for experimental protocol. Infarct size was calculated as percentage of the left ventricle.

### Western blot analysis

Western blot analysis was performed as described previously [13].

### Statistical analysis

Data are expressed as mean ± SD. Statistical calculations on infarct size data were performed using R (version 3.3.2). Group differences in infarct size were tested using multi-way ANOVA followed by Tukey post hoc test, one-way ANOVA followed by Tukey post hoc test or *t* test comparison as indicated; for details see Additional file 1. Hemodynamic data were analysed by two-way ANOVA for time and treatment effects. If an overall significance was found, comparisons between groups were made for each time point using ANOVA followed by Dunnett post hoc test with the control group as reference group. Time effects within each group were analysed by repeated measures ANOVA followed by two-tailed Dunnett post hoc test with the baseline value as the reference time point (Graph Pad Prism, v6.00, Graph Pad Software, La Jolla, Ca). p-values < 0.05 were considered significant (for single tests as well as for p-values adjusted for multiple testing). A sample size analysis was performed and yielded a group size of n = 10 as necessary to detect a difference in infarct size of 15% (power: 0.8; expected standard deviation: 0.08; α < 0.05, SigmaPlot 13, ANOVA sample size analysis).

## Results

Volunteer characteristics are shown in Table 2. No differences were observed in age between young male and female volunteers or between aged male and female volunteers. Body weight and height was higher in young and aged male volunteers compared to their respective age female groups.

**Table 2:** Demographic data

	n	Age (years)	Height (cm)	Body weight (kg)	BMI (kg/m^2^)
Young					
Male	10	23 ± 1	181 ± 5	74 ± 10	22.5 ± 2.1
Female	10	23 ± 1	168 ± 5*	62 ± 7*	21.9 ± 2.0
Old					
Male	10	69 ± 7	178 ± 6	84 ± 11	26.3 ± 3.1
Female	10	67 ± 4	162 ± 4*	61 ± 8*	22.8 ± 3.0*

Data are mean ± SD

*BMI* body mass index

* P < 0.05 vs. male

### Infarct size analysis

**Fig. 2:**
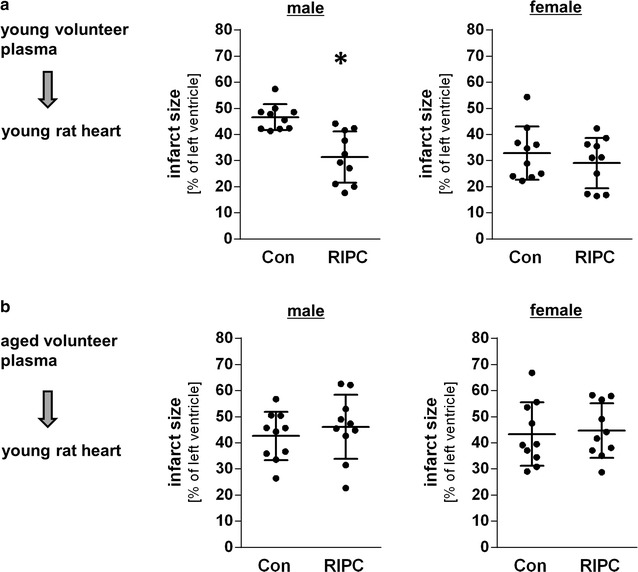
Age- and sex-dependent effects of RIPC plasma on infarct size in isolated hearts of young male rats. **a** Transfer of control and RIPC plasma of young male (left) and female (right) volunteers to isolated rat hearts. **b** Transfer of control and RIPC plasma of aged male (left) and female (right) volunteers to isolated rat hearts. Scatter plots show the infarct size in percent of the left ventricle. Data are presented as mean ± SD, n = 10 for all groups. *P < 0.05 vs. Con (multi-way (**a**) or one-way (**b**) ANOVA followed by Tukey’s post hoc test)

One way ANOVA showed that RIPC plasma from young male volunteers caused a relative infarct size reduction in young male rat hearts of 34% (31 ± 10% vs. 47 ± 5%; Fig. 2a) indicating a release of humoral factor(s) into the blood after RIPC. The infarct size in young male rat hearts after application of control plasma from young female volunteers was 33 ± 10%. RIPC plasma of young female did not initiate cardioprotection (Fig. 2a).

**Fig. 3:**
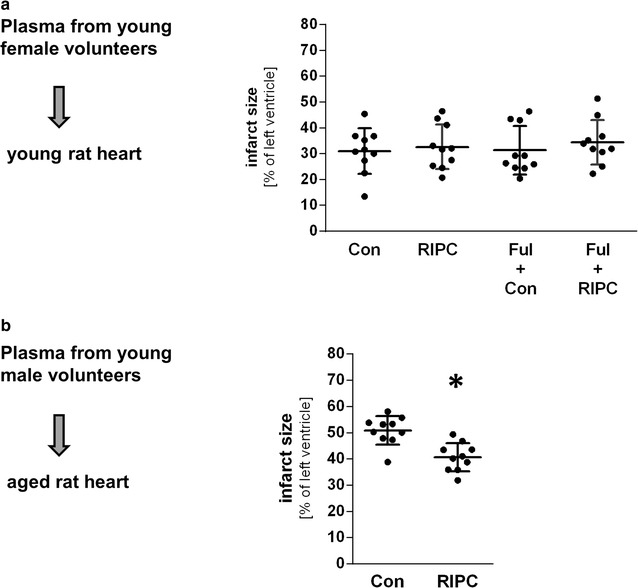
Effect of estrogen receptor blockade and cardioprotective potential of RIPC-plasma in the aged rat heart. **a** Effects of RIPC-plasma samples from young female volunteers on infarct size in young rat hearts in the absence or presence of estrogen receptor blocker fulvestrant (Ful). Scatter plot shows the infarct size in percent of the left ventricle. Data are presented as mean ± SD, n = 10 for all groups (one-way ANOVA followed by Tukey’s post hoc test). **b** Effects of RIPC-plasma samples from young male volunteers on infarct size in aged rat hearts. Scatter plot shows the infarct size in percent of left ventricle. Data are presented as mean ± SD, n = 10 for all groups. *P < 0.05 vs. Con (t-test comparison)

Despite the large numerical difference of the means values between infarct sizes from hearts treated with Con plasma from young female and male volunteers (male: 47 ± 5% vs. female: 33 ± 10%) the statistical test on difference did not reveal a significant difference (p = 0.06). Because a potential protective effect by female plasma could be mediated via estrogen receptor and mask a RIPC-effect by humoral factors, we tested the influence of estrogen receptors using the estrogen receptor inhibitor fulvestrant (Ful) on infarct size in young rat hearts perfused with plasma from young female volunteers (Con and RIPC). Infarct size after application of control plasma was 31 ± 9 and 33 ± 9% after RIPC plasma (Fig. 3a). Fulvestrant had no effect on infarct size compared to control group and RIPC group (Fig. 3a) indicating that a possible cardioprotective effect of humoral factor(s) after RIPC in young female plasma is not masked by a cardioprotective effect of estrogen receptor activation.

In contrast to RIPC plasma from young male volunteers, RIPC plasma from aged male volunteers had no effect on infarct size (Fig. 2b) clearly demonstrating an age-dependency of humoral factor release after RIPC. In addition, RIPC plasma from aged female volunteers did not reduce infarct size (Fig. 2b).

In order to ascertain that the effects of RIPC and age did not result from factor interaction we calculated multiway-ANOVA (including parameters RIPC, sex and age). The analysis confirmed that the estimated infarct size reduction of RIPC and young age both are 15% (p < 0.01).

Based on our results that RIPC releases cardioprotective humoral factors into the blood of young male volunteers as seen by a reduced infarct size after the transfer of RIPC plasma to young male rat hearts, we investigated the protective potency of the humoral factors to initiate cardioprotection in the aged rat heart. The application of RIPC plasma of young male volunteers initiated a relative infarct size reduction of 20% compared to control plasma (41 ± 5% vs. 51 ± 5%; *t* test; Fig. 3b).

### Hemodynamic measurements

At baseline, no differences in heart rate, left ventricular pressure and coronary flow were observed among the groups (Additional file 1: Table S1, Additional file 2: Table S2, Additional file 3: Table S3, Additional file 4: Table S4). No differences in coronary flow and left ventricular pressure were found between groups after application of blood plasma.

### Western blotting

**Fig. 4:**
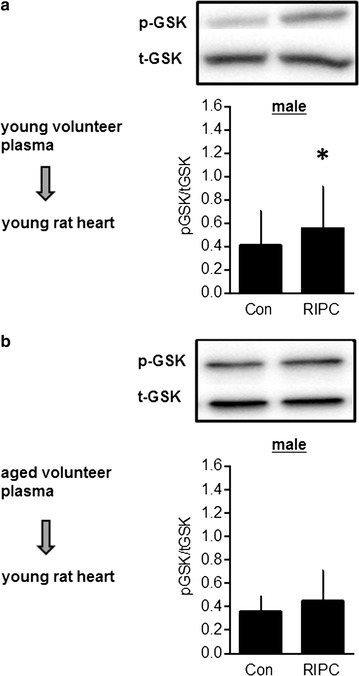
Age-dependent effects of RIPC plasma on GSK3β phosphorylation. Representative western blot analysis experiments and summarized data of GSK3β phosphorylation (Ser9) in heart tissue samples of young male rats after application of control (Con) and RIPC plasma of young (**a**) and aged volunteers (**b**). Summarized data presenting AVI (arbitrary units of average light intensity) are shown. Data are presented as mean ± SD, n = 10 for all groups. *P < 0.05 vs. Con (t-test comparison)

Additionally, potential intracardial signal transduction pathways—mediated by humoral factors—were investigated in consideration of potential age related differences after RIPC in male volunteers. RIPC plasma of young men increased phosphorylation of GSK3ß (0.56 ± 0.36 vs. Con: 0.41 ± 0.29; p < 0.05, Fig. 4 and Table 3), whereas plasma of old men had no effect on phosphorylation of GSK3ß. There were no differences in p-PLB and p-eNOS (Table 3).

**Table 3:** Western blot data (young and old male volunteers)

	n	Phospho protein/total protein			
		Young		Old	
		Con	RIPC	Con	RIPC
GSK3ß	10	0.41 ± 0.29	0.56 ± 0.36*	0.36 ± 0.13	0.45 ± 0.26
PLB	10	0.22 ± 0.14	0.24 ± 0.14	0.37 ± 0.18	0.34 ± 0.18
eNOS	10	0.78 ± 0.33	0.76 ± 0.28	0.71 ± 0.44	0.82 ± 0.53

Data are mean ± SD

*Con* control, *RIPC* remote ischemic preconditioning, *GSK3ß* glycogen synthase kinase-3ß, *PLB* phospholamban, *eNOS* endothelial nitric oxide synthase

* P < 0.05 vs. Con

## Discussion

Because of its non-invasive nature RIPC seems to be an optimal therapeutic strategy to protect the heart from I/R injury in a safe and easy-applicable way. There is strong evidence essentially from proof-of-concept studies for the potential benefit of RIPC in patients, e.g. significantly reduced the area under the curve of creatine kinase in patients with acute myocardial infarction [4], or significantly reduced serum troponin T levels after CABG surgery [14] or PCI [15]. Thielmann et al. even showed a significant better prognosis for patients after RIPC prior to elective CABG surgery [16]. However, two very recent large-scale trials (ERICCA and RIPHeart) failed to show a beneficial effect of RIPC [5, 6]. The underlying reason for this discrepancy is unclear, but several confounding factors are discussed. The choice of anesthetic regime influences RIPC as seen by a loss of protection using propofol [17]. In addition, other factors including medication, and individual patient characteristics as age and sex have been demonstrated to interfere with different cardioprotective strategies and might also affect RIPC. Theoretically, confounding factors might either affect the signal transduction pathways within the heart or the inter-organ communication, i.e. the signal transduction from the remote organ or tissue to the heart. The observation that the transfer of blood samples after RIPC to a naïve isolated heart initiates cardioprotection supports the concept of blood-borne humoral factors as an important signalling step of the inter-organ communication [18]. Although the exact molecular structure of the humoral factor(s) is still unknown, there is evidence that the factor(s) are hydrophobic, thermolabile low-molecular mass molecules (> 3.5; < 15 kDa) that circulate for up to 6 days [19–21]. Our result that the transfer of RIPC plasma from young male volunteers has a strong infarct-limiting effect is in line with previous findings that humoral factor(s) can be transferred to a naïve heart to initiate cardioprotection.

### Sex aspects of humoral factor release after RIPC

It has been demonstrated that sex-based differences in ischemia/reperfusion (I/R) injury exist [22]. Most studies show an increased tolerance of hearts from female animals compared to male hearts. The underlying reason for this difference might not be restricted to differences within the heart, because the results of the present study show a clear trend towards a lower infarct size (tested in male rat hearts) after the application of control plasma from young female volunteers control plasma from young male volunteers. This finding indicates that the blood plasma from young female volunteers might initiate cardioprotection per se. In addition to sex-differences in the I/R tolerance, also sex-differences in the efficacy of cardioprotective interventions have been described in some but not all studies [23–25]. Here, we demonstrate that RIPC plasma from young male volunteers reduced infarct size in hearts from male rats. In contrast, no infarct size reduction was observed after treatment with RIPC plasma from young female volunteers compared to hearts treated with control plasma from female volunteers. Because estrogen can initiate cardioprotection [26], it might have been feasible that a cardioprotective effect of RIPC plasma is masked by an infarct-limiting effect of estrogen receptor activation by young female plasma. However, the non-selective estrogen receptor antagonist fulvestrant had no influence on infarct size suggesting that the apparently smaller infarct size after application of young female plasma seems to be independent of estrogen receptor activation. Fulvestrant is a pure antiestrogen without estrogen agonistic side effects, which completely inhibits both estrogen receptors [27]. It has been demonstrated that the concentration of 100 nM fulvestrant is efficient to block cardiac effects of estrogen in the isolated rat heart [28]. Taken together, these data strongly suggest that sex-dependent differences in the release of humoral factor(s) after RIPC exist.

### Age-dependent aspects of humoral factor release after RIPC

There is some evidence that the protective effect of RIPC is age-dependent. Schmidt et al. demonstrated that RIPC rather impaired than improved cardiac function in neonatal rabbit and porcine hearts, respectively [29, 30]. In contrast, multiple cardioprotective strategies including ischemic preconditioning failed to initiate cardioprotection in the senescent heart [31]. Whether increasing age also affects the beneficial effects of RIPC it is debated controversially. A meta-analysis of Zhou et al. [9] showed a reduced incidence of acute kidney injury. In this study, the renoprotective effect was more pronounced in younger patients compared to older patients indicating an age-dependency. However, whether aging also affects the cardioprotective effect of RIPC is not completely understood. The recent large-scale clinical trials ERICCA and RIPHeart did not show a beneficial effect of RIPC, but the missing protection might be caused by other confounding factors than age, e.g. the choice of anesthesia [5, 6]. A retrospective analysis of a single-centre study from Thielmann et al. [16] did not offer evidence that increasing age affects the protective effect of RIPC [32]. However, because the study from Thielmann et al. was not powered for subgroup analysis, and more importantly, a control group with young patients is missing, the impact of increasing age on RIPC in patients remains unclear. On the other hand, we demonstrated recently that the infarct size reducing effect of RIPC was completely abolished in the aged rat heart [10]. In the present study, RIPC plasma of young male volunteers reduced infarct size in the isolated rat heart whereas blood plasma from aged male volunteers did not initiate cardioprotection. This finding identifies an age-dependency of the release of cardioprotective humoral factor(s) into the blood as possible underlying mechanism for an age-dependent loss of RIPC.

Next to this “defect in the inter-organ crosstalk” there might also be additional age-related alterations within the heart causing the abolished cardioprotective effect with increasing age. There is strong evidence that multiple cardioprotective strategies fail to reduce infarct size in the senescent heart [33]. However, it is reported that cardioprotection can be restored by chronic pre-treatments, e.g. caloric restriction [34], exercise [35] or administration of the radical scavenger tempol [36]. In addition, only a very low number of acute pharmacological treatments including blockade of the mitochondrial electron transport chain by amobarbital [37] or activation of mitochondrial calcium-sensitive potassium channels [38], have been described to initiate protection. As our findings demonstrate a release of humoral factors with strong infarct-limiting effect in RIPC plasma of young male volunteers, we tested the influence of these cardioprotective humoral factor(s) in hearts of aged male rats. Interestingly, RIPC plasma of young men reduced infarct size even in old rat hearts by 20% compared to control plasma. This indicates that the protective signalling mechanism that is targeted by the humoral factor(s) can be activated not only in the young but also in the aged rat myocardium.

### Signaling pathways

According to the results of our infarct size experiments, we investigated whether humoral factors in RIPC plasma of young male volunteers activates established signaling pathways from IPC within the isolated rat heart. Our results show that RIPC plasma of young male volunteers increased phosphorylation of GSK3ß in rat myocytes. GSK3ß is a well-known downstream target of the reperfusion injury salvage kinase (RISK) pathway and its phosphorylation results in inhibition of the mitochondrial permeability transition pore (mPTP), which is closely linked to cardioprotection [39]. Our data show that this protective signaling pathway is regulated after application of RIPC plasma to isolated hearts, i.e. exclusively by humoral factors. In contrast, GSK3ß phosphorylation was not altered after application of RIPC plasma from old men. This finding further confirms our results from infarct size experiments that no humeral factor is released in old volunteers after RIPC.

Recently, we could demonstrate that activation of PKA is involved in cardioprotective signaling pathways, and furthermore, that the protective effect of PKA activation is age-dependent in aged rat hearts in vivo [38]. Our results that humoral factor transfer to the isolated heart does not affect PLB phosphorylation, which was assessed as marker for protein kinase A activity, suggest that PKA activation might not be involved in RIPC induced cardioprotection. In addition, no differences in phosphorylation of eNOS were detected suggesting that the NO-PKG pathway, that is involved in cardioprotective signaling pathways [40], is not involved in cardioprotection by RIPC. However, Slagsvold et al. showed in patients undergoing CABG surgery that RIPC did not increase myocardial p-GSK3ß [41]. The reason for this discrepancy remains unclear, but a possible explanation might be a difference in the time point of tissue sampling. In addition, Slagsvold et al. demonstrated that RIPC increases Akt phosphorylation, which is located directly upstream of GSK3ß indicating activation of this cardioprotective signaling pathway. Taken together, our findings suggest that humoral factors after RIPC lead to cardioprotection by activation of the RISK pathway.

## Conclusion

In summary, our results show that humoral factor(s) are released into the blood after RIPC in young male humans. However, the release of these humoral factor(s) is age dependent. Our finding that RIPC in women did not initiate the release of humoral factor(s) demonstrates the existence of sex-differences in humoral factor release. In addition, the humoral factor(s) have the potency to initiate cardioprotection also in the aged heart.

### Limitations

The results of the present study have to be interpreted within the light of several study limitations. First, the understanding of the mechanistic signalling of RIPC remains unresolved, e.g. the molecular nature of the humoral factor(s) as well as its receptor is unknown. Therefore, the age- and sex-dependency of the humoral factor(s) release can be identified only indirectly by measuring the infarct size reducing effects of the different plasma samples. In this line, we cannot exclude the possibility that the humoral factor(s) are not consistent between different age- or sex-groups. Second, because we did not investigate different plasma concentrations in our bioassay it remains unknown whether a dose-dependency exist. Here, a perfusion rate of 0.5% of the coronary flow was used because higher perfusion rates with human blood plasma affected left ventricular function. Therefore, we cannot exclude a possible infarct size reducing effect at higher perfusion rates, and it might be possible that humoral factor(s) are also released into the blood of old volunteers after RIPC, but only to a lesser extend as in young volunteers.

### Future directions

This study clearly identifies both age and sex as confounding factors that interfere with the protective effects of RIPC, and therefore, will help to explain existing differences between the results of both experimental and clinical studies investigating the cardioprotective potential of RIPC. However, for a complete mechanistic insight is the identification of the humoral factor(s) essential. More importantly, because the humoral factor(s) have the potential to initiate protection in the aged heart, the identification of the cardiac receptor is of great interest as pharmacological target. Therefore, future studies aiming to identify the molecular structure of humoral factor(s) as well as the cardiac receptor are required.

## Additional files

**Additional file 1: Table S1.** Hemodynamic variables (plasma from young volunteers).

**Additional file 2: Table S2.** Hemodynamic variables (plasma from aged volunteers).

**Additional file 3: Table S3.** Hemodynamic variables (plasma from young female volunteers).

**Additional file 4: Table S4.** Hemodynamic variables (plasma from young male volunteers, aged rat hearts).

## Abbreviations

RIPC: remote ischemic preconditioning
CVD: cardiovascular disease
I/R: ischemia and reperfusion
TTC: 2,3,5-triphenyltetrazolium chloride
CABG: coronary artery bypass graft
