# Supplementary material for: The release of cardioprotective humoral factors after remote ischemic preconditioning in humans is age- and sex-dependent
Source: J Transl Med. 2018 Apr 27;16:112. doi: 10.1186/s12967-018-1480-0 (PMC5921545; doi:10.1186/s12967-018-1480-0)
Supplement: Supplementary file 1 — Additional file 1: Table S1. Hemodynamic variables (plasma from young volunteers). [file 12967_2018_1480_MOESM1_ESM.docx]

**Table S1: Hemodynamic variables (plasma from young volunteers)**

| Plasma | Group | Baseline | PC | Reperfusion | |
| --- | --- | --- | --- | --- | --- |
|  |  |  |  | 30 | 60 |
| *Heart Rate (bpm)* | | | | | |
| Male | Con | 320 ± 42 | 302 ± 50 | 312 ± 40 | 285 ± 58 |
|  | RIPC | 334 ± 71 | 316 ± 83 | 304 ± 52 | 295 ± 48 |
| Female | Con | 345 ± 61 | 331 ± 41 | 268 ± 90* | 278 ± 61* |
|  | RIPC | 311 ± 50 | 309 ± 42 | 306 ± 38 | 282 ± 36 |
| *Phasic LVP (mmHg)* | | | | | |
| Male | Con | 122 ± 18 | 89 ± 20* | 23 ± 11* | 31 ± 8* |
|  | RIPC | 113 ± 28 | 84 ± 28* | 27 ± 15* | 30 ± 8* |
| Female | Con | 113 ± 21 | 90 ± 20* | 27 ± 14* | 29 ± 9* |
|  | RIPC | 125 ± 14 | 102 ± 24* | 23 ± 12* | 32 ± 9* |
| *CF (ml*min^-1^)* | | | | | |
| Male | Con | 19 ± 3 | 15 ± 2* | 10 ± 1* | 9 ± 2* |
|  | RIPC | 17 ± 4 | 13 ± 4* | 9 ± 3* | 8 ± 3* |
| Female | Con | 19 ± 4 | 15 ± 4* | 9 ± 2* | 8 ± 2* |
|  | RIPC | 17 ± 3 | 15 ± 3 | 10 ± 3* | 9 ± 2* |

Data are mean±SD.

Con = control; PC = preconditioning; RIPC = remote ischemic preconditioning.

*P<0.05 vs. baseline.
