# Supplementary material for: The release of cardioprotective humoral factors after remote ischemic preconditioning in humans is age- and sex-dependent
Source: J Transl Med. 2018 Apr 27;16:112. doi: 10.1186/s12967-018-1480-0 (PMC5921545; doi:10.1186/s12967-018-1480-0)
Supplement: Supplementary file 2 — Additional file 2: Table S2. Hemodynamic variables (plasma from aged volunteers). [file 12967_2018_1480_MOESM2_ESM.docx]

**Table S2: Hemodynamic variables (plasma from aged volunteers)**

| Plasma | Group | Baseline | PC | Reperfusion | |
| --- | --- | --- | --- | --- | --- |
|  |  |  |  | 30 | 60 |
| *Heart Rate (bpm)* | | | | | |
| Male | Con | 330 ± 38 | 313 ± 31 | 334 ± 20 | 297 ± 19* |
|  | RIPC | 328 ± 34 | 318 ± 42 | 313 ± 28 | 324 ± 29 |
| Female | Con | 322 ± 41 | 308 ± 38 | 318 ± 24 | 299 ± 25 |
|  | RIPC | 305 ± 32 | 304 ± 24 | 310 ± 24 | 320 ± 46 |
| *Phasic LVP (mmHg)* | | | | | |
| Male | Con | 128 ± 17 | 102 ± 24* | 14 ± 8* | 21 ± 7* |
|  | RIPC | 121 ± 19 | 102 ± 26* | 13 ± 7* | 17 ± 8* |
| Female | Con | 131 ± 16 | 104 ± 20* | 15 ± 7* | 18 ± 6* |
|  | RIPC | 129 ± 9 | 106 ± 22* | 13 ± 8* | 16 ± 8* |
| *CF (ml*min^-1^)* | | | | | |
| Male | Con | 15 ± 3 | 10 ± 3* | 9 ± 4* | 7 ± 2* |
|  | RIPC | 13 ± 2 | 11 ± 2 | 9 ± 4* | 7 ± 2* |
| Female | Con | 15 ± 3 | 10 ± 2* | 9 ± 5* | 7 ± 2* |
|  | RIPC | 14 ± 2 | 10 ± 2* | 8 ± 4* | 7 ± 4* |

Data are mean±SD.

Con = control; PC = preconditioning; RIPC = remote ischemic preconditioning.

*P<0.05 vs. baseline.
