# Supplementary material for: The release of cardioprotective humoral factors after remote ischemic preconditioning in humans is age- and sex-dependent
Source: J Transl Med. 2018 Apr 27;16:112. doi: 10.1186/s12967-018-1480-0 (PMC5921545; doi:10.1186/s12967-018-1480-0)
Supplement: Supplementary file 3 — Additional file 3: Table S3. Hemodynamic variables (plasma from young female volunteers). [file 12967_2018_1480_MOESM3_ESM.docx]

**Table S3: Hemodynamic variables (plasma from young female volunteers)**

| Plasma | | Group | Baseline | PC | Reperfusion | |
| --- | --- | --- | --- | --- | --- | --- |
|  | |  |  |  | 30 | 60 |
| *Heart Rate (bpm)* | | | | | | |
| Female | Con | | 332 ± 48 | 322 ± 29 | 349 ± 64 | 303 ± 112 |
|  | RIPC | | 343 ± 37 | 348 ± 35 | 313 ± 55 | 310 ± 35 |
|  | Ful+Con | | 343 ± 33 | 321 ± 37 | 293 ± 41 | 285 ± 38 |
|  | Ful+RIPC | | 311 ± 56 | 307 ± 44 | 263 ± 67 | 260 ± 35 |
| *Phasic LVP (mmHg)* | | | | | | |
| Female | Con | | 140 ± 26 | 111 ± 21* | 23 ± 10* | 28 ± 10* |
|  | RIPC | | 137 ± 23 | 110 ± 22* | 20 ± 13* | 25 ± 7* |
|  | Ful+Con | | 134 ± 15 | 115 ± 13* | 20 ± 10* | 26 ± 11* |
|  | Ful+RIPC | | 130 ± 23 | 113 ± 18* | 20 ± 8* | 25 ± 7* |
| *CF (ml*min^-1^)* | | | | | | |
| Female | Con | | 17 ± 3 | 13 ± 4* | 11 ± 5* | 11 ± 6* |
|  | RIPC | | 16 ± 4 | 13 ± 3 | 13 ± 5* | 12 ± 4* |
|  | Ful+Con | | 18 ± 4 | 14 ± 4* | 13 ± 5* | 9 ± 2* |
|  | Ful+RIPC | | 16 ± 4 | 14 ± 4 | 11 ± 4* | 10 ± 4* |

Data are mean±SD.

Con = control; PC = preconditioning; RIPC = remote ischemic preconditioning; Ful = fulvestrant. *P<0.05 vs. baseline.
