# Supplementary material for: The release of cardioprotective humoral factors after remote ischemic preconditioning in humans is age- and sex-dependent
Source: J Transl Med. 2018 Apr 27;16:112. doi: 10.1186/s12967-018-1480-0 (PMC5921545; doi:10.1186/s12967-018-1480-0)
Supplement: Supplementary file 4 — Additional file 4: Table S4. Hemodynamic variables (plasma from young male volunteers, aged rat hearts). [file 12967_2018_1480_MOESM4_ESM.docx]

**Table S4: Hemodynamic variables (plasma from young male volunteers, aged rat hearts)**

| Plasma | Group | Baseline | PC | Reperfusion | |
| --- | --- | --- | --- | --- | --- |
|  |  |  |  | 30 | 60 |
| *Heart Rate (bpm)* | | | | | |
| Male | Con | 255 ± 46 | 228 ± 47 | 224 ± 74 | 223 ± 54 |
|  | RIPC | 261 ± 28 | 252 ± 31 | 234 ± 70 | 207 ± 59* |
| *Phasic LVP (mmHg)* | | | | | |
| Male | Con | 131 ± 17 | 118 ± 21 | 19 ± 12* | 19 ± 14* |
|  | RIPC | 125 ± 18 | 112 ± 18 | 14 ± 8* | 13 ± 8* |
| *CF (ml*min^-1^)* | | | | | |
| Male | Con | 23 ± 4 | 20 ± 4 | 16 ± 5* | 14 ± 5* |
|  | RIPC | 26 ± 4 | 24 ± 8 | 20 ± 8* | 18 ± 6* |

Data are mean±SD.

Con = control; PC = preconditioning; RIPC = remote ischemic preconditioning.

*P<0.05 vs. baseline.
